# Tethered spinal cord tension assessed via ultrasound elastography in computational and intraoperative human studies

**DOI:** 10.1038/s43856-023-00430-6

**Published:** 2024-01-05

**Authors:** Max J. Kerensky, Abhijit Paul, Denis Routkevitch, Andrew M. Hersh, Kelley M. Kempski Leadingham, A. Daniel Davidar, Brendan F. Judy, Joshua Punnoose, Autumn Williams, Avisha Kumar, Kurt Lehner, Beth Smith, Jennifer K. Son, Javad R. Azadi, Himanshu Shekhar, Karla P. Mercado-Shekhar, Nitish V. Thakor, Nicholas Theodore, Amir Manbachi

**Affiliations:** 1 Department of Biomedical Engineering, Johns Hopkins University School of Medicine, Baltimore, MD USA; 2 HEPIUS Innovation Laboratory, Johns Hopkins University School of Medicine, Baltimore, MD USA; 3 Discipline of Biological Engineering, Indian Institute of Technology Gandhinagar, Gujarat, India; 4 Department of Neurosurgery, Johns Hopkins University School of Medicine, Baltimore, MD USA; 5 Department of Electrical and Computer Engineering, Johns Hopkins University, Baltimore, MD USA; 6 Department of Radiology and Radiological Science, Johns Hopkins University School of Medicine, Baltimore, MD USA; 7 Discipline of Electrical Engineering, Indian Institute of Technology Gandhinagar, Gujarat, India; 8 Department of Neurology, Johns Hopkins University School of Medicine, Baltimore, MD USA; 9 Department of Orthopaedic Surgery, Johns Hopkins University School of Medicine, Baltimore, MD USA; 10 Department of Pediatrics, Johns Hopkins University School of Medicine, Baltimore, MD USA; 11 Department of Anesthesiology and Critical Care Medicine, Johns Hopkins University School of Medicine, Baltimore, MD USA; 12 Department of Mechanical Engineering, Johns Hopkins University, Baltimore, MD USA

**Keywords:** Spinal cord diseases, Ultrasound, Diagnostic markers, Ultrasonography, Paediatric research

## Abstract

**Background:**

Tension in the spinal cord is a trademark of tethered cord syndrome. Unfortunately, existing tests cannot quantify tension across the bulk of the cord, making the diagnostic evaluation of stretch ambiguous. A potential non-destructive metric for spinal cord tension is ultrasound-derived shear wave velocity (SWV). The velocity is sensitive to tissue elasticity and boundary conditions including strain. We use the term Ultrasound Tensography to describe the acoustic evaluation of tension with SWV.

**Methods:**

Our solution Tethered cord Assessment with Ultrasound Tensography (TAUT) was utilized in three sub-studies: finite element simulations, a cadaveric benchtop validation, and a neurosurgical case series. The simulation computed SWV for given tensile forces. The cadaveric model with induced tension validated the SWV-tension relationship. Lastly, SWV was measured intraoperatively in patients diagnosed with tethered cords who underwent treatment (spinal column shortening). The surgery alleviates tension by decreasing the vertebral column length.

**Results:**

Here we observe a strong linear relationship between tension and squared SWV across the preclinical sub-studies. Higher tension induces faster shear waves in the simulation (*R*^2^ = 0.984) and cadaveric (*R*^2^ = 0.951) models. The SWV decreases in all neurosurgical procedures (*p* < 0.001). Moreover, TAUT has a c-statistic of 0.962 (0.92-1.00), detecting all tethered cords.

**Conclusions:**

This study presents a physical, clinical metric of spinal cord tension. Strong agreement among computational, cadaveric, and clinical studies demonstrates the utility of ultrasound-induced SWV for quantitative intraoperative feedback. This technology is positioned to enhance tethered cord diagnosis, treatment, and postoperative monitoring as it differentiates stretched from healthy cords.

## Plain language summary

Tethered spinal cord syndrome occurs when surrounding tissue attaches to and causes stretching across the spinal cord. People with a tethered cord can experience weakness, pain, and loss of bladder control. Although increased tension in the spinal cord is known to cause these symptoms, evaluating the amount of stretching remains challenging. We investigated the ability of an ultrasound imaging approach to measure spinal cord tension. We studied our method in a computer simulation, a benchtop validation model, and in six people with tethered cords during surgery that they were undergoing to reduce tension. In each phase, the approach could detect differences between stretched spinal cords and spinal cords in a healthy state. Our method could potentially be used in the future to improve the care of people with a tethered cord.

## Abstract (web summary)

Kerensky et al. quantify tension across human spinal cords in computational simulations, a cadaveric benchtop model, and a neurosurgical case series. Their direct methodology successfully differentiates stretched spinal cords from healthy states in all sub-studies.

## Introduction

The spinal cord floats relatively freely in cerebrospinal fluid, especially in the lower back (lumbosacral region)^1^. However, excessive stretching of the cord is reported to be present in an estimated 1 out of every 1000-4000 births and can develop or progress over a lifetime^2–6^. This tension causes neurological deficits^7–10^. Tethered cord syndrome (TCS) is the commonly used medical term to describe suspected tension-induced dysfunctions which can include pain, muscle weakness, and loss of bladder and bowel control^11–14^. Unfortunately, this tension is often only suspected, as clinicians lack a methodology to quantify the amount of force across the bulk of the spinal cord during the diagnostic and treatment processes of TCS.

Without a force metric, doctors must evaluate a patient’s spinal cord tension with secondary, indirect information sources which include symptoms and anatomical correlations. Patients with indicators of TCS can be healthy while others without obvious indications might have excess tension. The signs, presentations, and causes of spinal cord tension are heterogeneous^15–18^. Some cases might not have obvious anatomical indicators via conventional workup using either magnetic resonance imaging (MRI) or ultrasound in the prenatal and neonatal populations^19^. A diagnosis of occult TCS is given when classical indicators are absent, but tethering is believed to be present^20,21^. Most cases, however, have indications that can range from a stigmata on the skin (a sacral dimple or hairy patch) to a thickened filum terminale (a fattier, fibrous band at the end of the spinal cord)^22–25^. Electrophysiological signatures of TCS have also been emerging^26,27^. Although these collective features and symptoms currently allow for a clinical diagnosis of TCS, one cannot definitively conclude tension as the cause of symptoms.

The relative length of the spinal cord as an indicator of stretch can also be misleading. In the clinic, relative spinal cord length is calculated using the position of the conus medullaris, the tapered portion of the cord. The increased length is used as a surrogate for tension^6,28^. However, the normal anatomical variability of spinal cord lengths adds uncertainty to this metric^29–31^. A more direct study of suspected tension involved stretching the filum terminale to gauge elasticity and, thus, possible TCS^32^. However, tension across the bulk of the cord remained unknown. Stretching this sensitive region is not clinically viable as it presents undue risks of inducing additional deficits. So, the question remains: how can tension across the spinal cord be safely quantified?

The two foremost promising candidate technologies to safely evaluate tension are MRI- and ultrasound-based approaches as they permit non-destructive visualizations of the spinal cord. Ultrasound approaches are considered particularly favorable due to their increased accessibility, lower cost, intraoperative availability, and relative ease of use, especially in newborn populations^33–35^.

Elastography, an imaging modality, has emerged as a reliable diagnostic tool for measuring tissue stiffness. Ultrasound elastography imaging can be conducted with new stretchable ultrasonic patches or with commercially available products already adopted in hospitals^36^. Strain elastography and shear wave elastography (SWE) are the two major types of ultrasound elastography. SWE is particularly appealing for spinal cord imaging because it is quantitative and does not require organ compression^37^. In this technique, an acoustic impulse is delivered into the targeted tissue, either over a small field of view (point SWE) or over a large field of view (2D-SWE). The impulse causes shear waves to propagate perpendicular to the direction of the impulse; in our case, shear waves can be tracked along the length of the cord. A faster shear wave velocity (SWV) is associated with an increase in tissue stiffness. This phenomenon is utilized across the body for multiple diagnostic purposes, most commonly to assess hepatic fibrosis or locate tumors^38–41^. While SWV has been modeled in soft tissue materials with varying fiber alignments and strain, its utility in spinal cord tension quantification has yet to be investigated^42–44^. Recent shear wave elastography studies have successfully quantified compression in the spinal cord^45,46^. Although this approach supplements clinical insights, deformation from a spinal cord injury is often visualizable with conventional imaging (e.g., MRI, computed tomography, or B-mode ultrasound imaging)^47^. Elastography would be especially useful if it could provide insights that are not obtainable through conventional techniques^48^. As emphasized, neurosurgeons lack a direct diagnostic tool for spinal cord tension, and an elastography-based approach might resolve this clinical gap.

We hypothesized that ultrasound-based SWV measurements could be a reliable, quantitative metric of spinal cord tension, aiding in TCS diagnoses and surgical interventions. We call our proposed methodology, Tethered cord Assessment with Ultrasound Tensography (TAUT). TAUT is grounded in the theoretical SWV changes resulting from applied tensile forces (not solely the inherent elasticity)^42,49,50^. The underlying technology has been established in other tissues including tendons; in this context, it is often called shear wave tensiometry^49,51,52^. We use the term Tensography to minimize confusion with the classical implications of tensiometry (i.e., interfacial or surface tension). To investigate the utility of TAUT, the relationship between SWV and spinal cord tension was first modeled computationally using finite element simulations. After characterizing the SWV-tension relationship in a virtual spinal cord, a proof-of-concept cadaveric model was used to validate TAUT in a controlled setting on human tissue. Finally, its clinical value was demonstrated in a human intraoperative setting. TAUT was used in posterior vertebral column subtraction osteotomy (PVCSO) procedures, in which patients with suspected TCS underwent shortening of the vertebral column to alleviate tension^53,54^. Here, we report a quantitative tensile force metric for the differentiation of healthy and stretched spinal cords.

## Methods

**Fig. 1:**
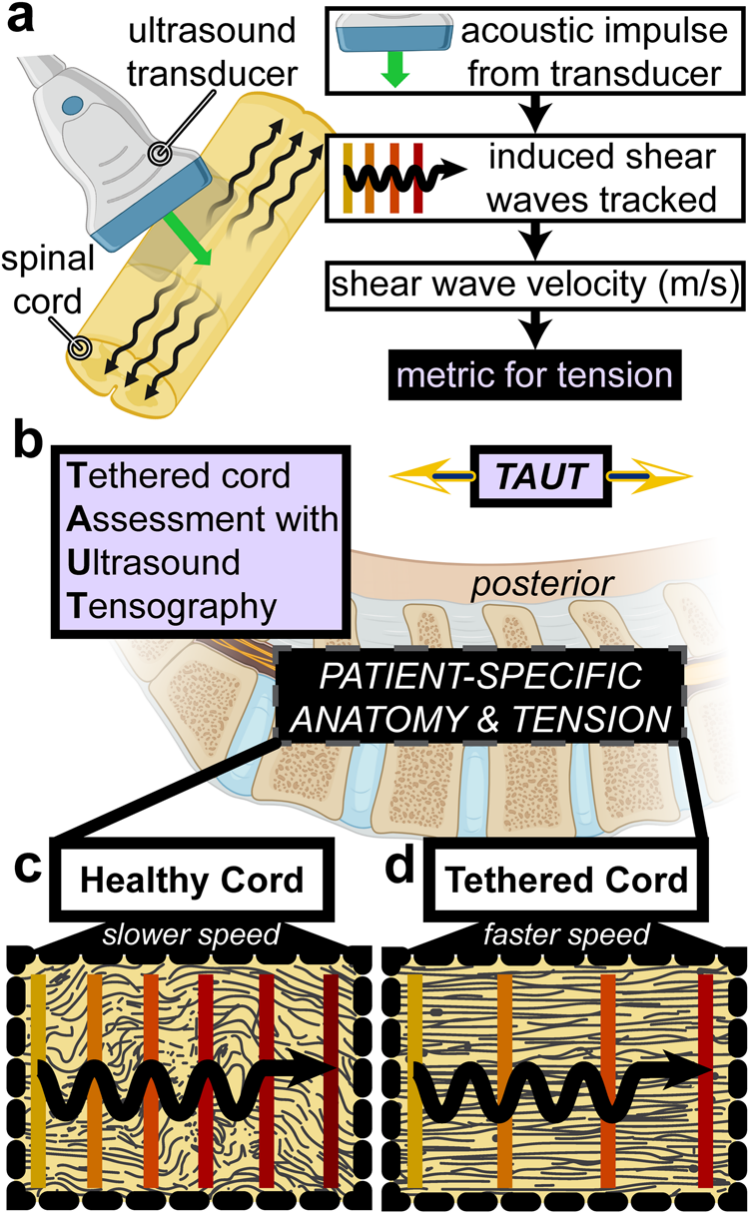
Principles and hypothesized utility of Tethered cord Assessment with Ultrasound Tensography (TAUT) to quantify spinal cord tension. **a** An ultrasound transducer is positioned above the spinal cord (without direct contact), inducing and tracking shear waves in the sagittal plane. Shear wave velocity (SWV) is dependent on both intrinsic and boundary conditions of an organ, including an externally applied force. We hypothesized that the SWV across a stretched spinal cord is faster than that of its free-floating equivalent. **b** There is anatomical variability among patients with suspected spinal cord conditions. Consequently, the amount of tension across cords can vary from healthy to excessive stretching as present in tethered cord syndrome (TCS). Acquiring ultrasound-induced SWV and relating it to tension (an approach we termed TAUT) might aid clinical evaluations. **c** The theorized resting microstructure (illustrated in dark gray) and the corresponding shear wave propagation are depicted in a healthy spinal cord and **d** in a tethered spinal cord. The microstructure (based on Brieg’s 1972 stains^79^) and loading of a spinal cord are hypothesized to affect SWV. Parts a and b were partially created with BioRender.com.

Three clinically relevant sub-studies evaluated the effects of tension across the spinal cord on SWV: (1) a finite element simulation, (2) a proof-of-concept human cadaveric benchtop experiment, and (3) a series of 6 human neurosurgical cases. The translational investigation examines the capabilities of TAUT (as described in Fig. 1) in TCS applications.

### Finite element simulation

**Fig. 2:**
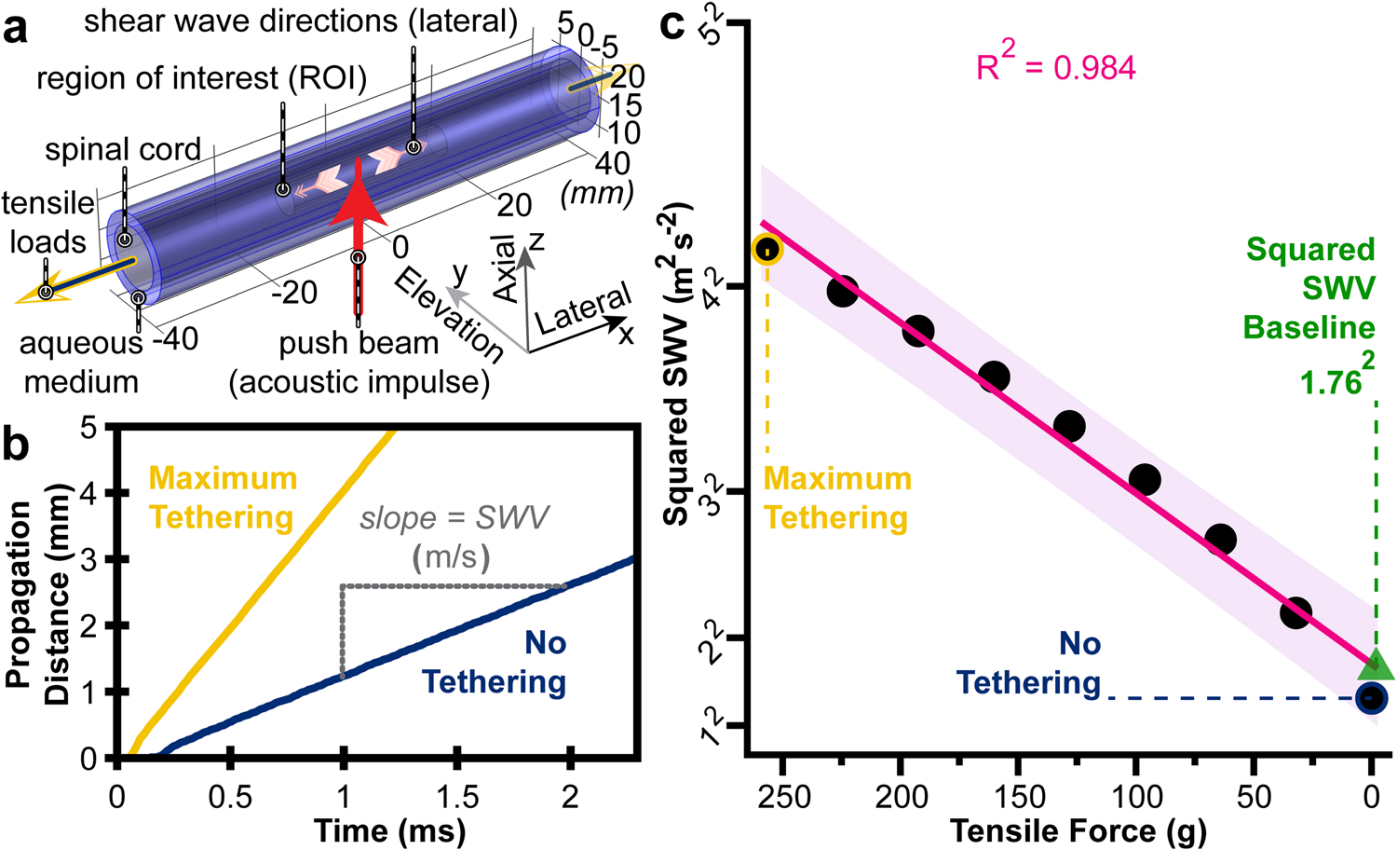
A finite element simulation of SWV in the spinal cord under incremental tensile loads. **a** An illustration of the model configuration is shown. The term lateral is utilized in its traditional ultrasound verbiage, which counterintuitively corresponds to the anatomical cranial-caudal axis. Similarly, the term elevation anatomically corresponds to the medial-lateral axis. Ultrasound induced and tracked shear wave propagation through the spinal cord at fixed tensile loads. **b** A smoothed trace (maximum amplitude) of the shear wave wavefront was plotted for the maximum applied load (256 g) in yellow and the cord without an applied load (0 g) in blue. **c** The squared SWV-tension relationship was plotted with a linear regression fit and prediction band (95%) in magenta. The squared SWV baseline of 3.108 m^2^ s^−2^ equates to a SWV simulation baseline of 1.76 m/s.

A cylindrical (5 mm radius) spinal cord model was constructed with an ultrasound transducer located 15 mm below its center as depicted in Fig. 2a. One-fourth of the respective domain was used in the simulation, symmetric along the lateral (x) and elevation (y) directions. Shear waves were induced from the transducer and tracked along the virtual cord at different tensile loads. The commercial COMSOL Multiphysics® software was used to establish the tensile loading and responses across the cord^55^. The simulation parameters were based on (and extrapolated from) known values (Supplementary Table 1). Besides the applied forces, all parameters were held constant across the simulations, isolating the tension-SWV relationship.

In line with previous studies, the spinal cord was assumed to be homogeneous, linear elastic, and orthotropic [i.e., mechanical properties in its lateral (lengthy) direction differed from the two orthogonal, radial directions]^56,57^. The transducer was acoustically coupled to the cord by an isotropic water medium. The aqueous medium surrounding the spinal cord mimics the fluid-solid interface. A uniform high-density hexahedral mesh (element size 0.2 mm) was used at the cylindrical-shaped (2.5 mm radius) region of interest (ROI) defined by the acoustic impulse location and shear wave tracking field. A relatively coarser non-uniform tetrahedral mesh (element size ranging from 0.2 mm to 0.8 mm) was set for the rest of the domain to reduce the computational complexity^55^. These meshes enable independent and reliable shear wave tracking at the ROI. Throughout the entire simulation, an absolute computational tolerance was set as 10^−6^ to ensure time step independence. The simulations were conducted on a workstation with an AMD Ryzen 9 5950×16-core processor and 96 GB RAM.

The pre-stretch condition was implemented by setting a static uniaxial tensile boundary load at the left and right sides of the spinal cord domain^58^. The acoustic impulse push beam from the transducer was applied on the center of the cord with a 1000 W cm^−2^ spatial peak pulse average intensity^59,60^. The induced shear waves were simulated for a propagation period of 2.3 ms under each pre-stretched condition to track the local effects without interference. The outer surfaces of both the cylinders in the domain (Fig. 2a) were constrained along normal directions to the boundary. The computational complexity was reduced by the choice of simulation domain lateral size and shear wave propagation period.

The acoustic intensity profile of the push beam in 3-dimensional space was simulated using Field II in MATLAB (version R2021a) with linear array transducer parameters listed in Supplementary Table 1^61,62^. The acoustic impulse was applied as a body load in the finite element model, as described in Palmeri et al. and clarified in our Supplementary Methods^55^. Next, the spatiotemporal profile of the propagating shear wave at each axial depth in the spinal cord was generated. The velocity of the shear wave was computed based on the “time to peak” velocity method^63^. This approach calculates the SWV by measuring the time at which the peak of the shear wave front propagates to certain distances. The slope of the best-fit line of the propagation distance as a function of time equates to the SWV, as shown in Fig. 2b.

To study the effects of tension on SWV, 9 incremental boundary loads (i.e., stresses) were applied to the virtual cord. The stresses ranged from 0 to 32 kPa at 4 kPa increments, modified from a recent biomechanical spinal cord study to overlap with our cadaveric benchtop model^64^. These values approximately equate to 0, 32, 64, 96, 128, 160, 192, 224, and 256-gram loads across the spinal cord. The maximum external load of 256 grams was chosen to fully encompass the tensile range of the cadaveric study.

### Cadaveric Benchtop Study

#### Experimental setup of the Benchtop Study

**Fig. 3:**
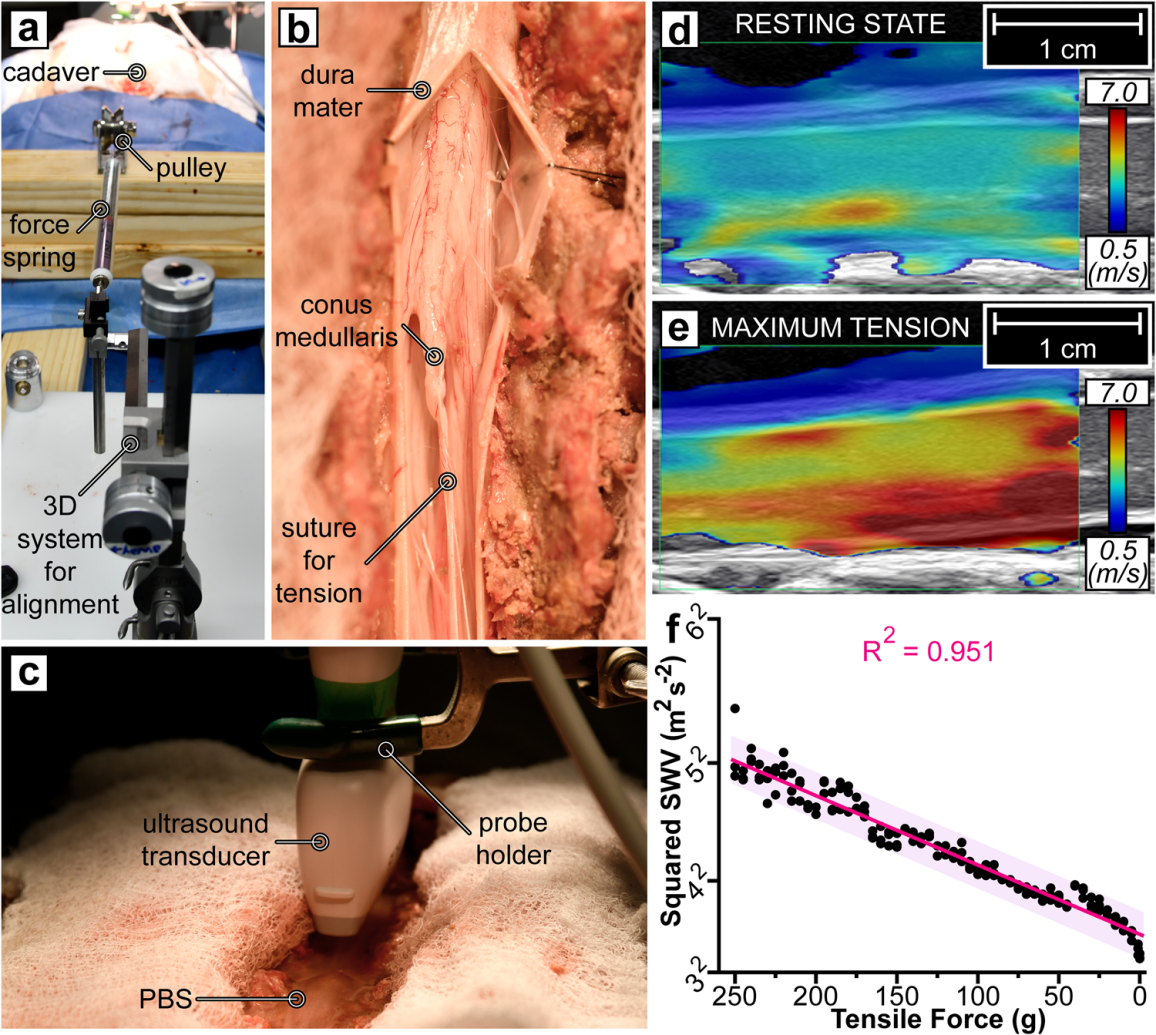
Proof-of-concept cadaveric model of ultrasound-induced shear waves as a metric for spinal cord tension. **a** A unidirectional tensile force was applied to the cadaveric spinal cord. The 3D stereotactic arm helped position the force gauge spring in-plane with the cord. A suture connected the spring to the spinal cord, passing over a pulley for stability and reliability. **b** The suture was anchored to the conus medullaris (tapered end of the spinal cord) with a Roman sandal-style knot. **c** The dorsal cavity was filled with phosphate-buffered saline (PBS) and the ultrasound probe was fixed in place by a probe holder. **d** The SWV heatmap of the spinal cord at rest and **e** under maximum tension of (250 g) is shown. The dura mater was not directly stretched. **f** From relaxation (0 and 1 g), the spinal cord was pulled at 5-increments (from 5 to 250 g), pausing for three ultrasound acquisitions at each tensile force. The squared SWV values were plotted with a linear regression best-fit line and prediction band (95%) in magenta. The values on the *x*-axis descend to visually align with the other sub-studies and clinical application of reducing tension.

A TCS model was created in an adult human cadaver with no known history of prior surgeries and devoid of confounding spine pathology (State Anatomy Board of Maryland, pre-registered donation with informed consent for medical research). This study was carried out in accordance with the authorization and purchasing policies of Johns Hopkins and Maryland State law, which did not require prior ethical approval by an ethical committee for the benchtop study. Creating a physical benchtop TCS model enabled a controlled investigation into the tensile force-SWV relationship in the spinal cord prior to intraoperative testing (Fig. 3a–c). The cadaver was positioned prone on a Jackson table with the head in a fixed position. The following describes the surgical approach to expose the cord for direct tensile stretching and corresponding ultrasound imaging: a continuous incision was created from the tenth thoracic (T10) vertebral level to the sacrum, followed by a subperiosteal, tissue-clearing dissection. A wide laminectomy was performed from T10 to the fifth lumbar (L5) vertebral level along with a partial sacrectomy. A durotomy from T10 to the second sacral (S2) vertebral level exposed the spinal cord, enabling direct tensile loading on the bulk of the cord.

Tension was induced by tying a Roman sandal-style suture around the conus medullaris and passing the other end over a pulley system latched onto a spring scale (2.5 N/250 g Premium Dynamometer, Eisco Labs, Victor, NY)^65^. A 3-dimensional stereotactic arm was used to position the spring parallel to and in line with the spinal cord without tissue obstruction. Excess slack in the suture was removed using the stereotactic arm.

#### Cadaveric ultrasound acquisition and analysis

The entire dorsal cavity was flooded with phosphate-buffered saline (PBS) solution to provide acoustic coupling for ultrasound imaging. PBS was utilized instead of ultrasound gel due to its ability to fill hard-to-access gaps underneath the dura. The ultrasound images were collected on a Canon Aplio i800 in 2D-SWE mode with a Canon i18LX5 transducer (Canon Medical Systems, Otawara, Japan). The ultrasound system simultaneously captured B-mode (grayscale anatomical view) and 2D-SWE data. The 2D-SWE propagation map is a representation of the shear wave wavefront overtime on top of the B-mode image. The 2D-SWE heatmap displays colors corresponding to the SWV at locations in the anatomical view. The probe was oriented along the sagittal length of the cord without direct contact. The transducer was fixed by a probe holder rostral to the resting conus, in a region with dura intact. Trapped air bubbles were visualized underneath the dura using ultrasound and were removed from the region of interest via a pipette. Three shear wave image acquisitions were obtained at each loading condition. To minimize a potential initial spring error, baseline acquisitions were recorded at both 0 and 1 gram. Thereafter, the force on the cord was set from 5 to 250 g in 5-g increments.

The mean SWV through the spinal cord in each image acquisition was extracted using the built-in region of interest trace tool on the Canon system. While absolute cadaveric SWV values do not typically align with clinical data, the SWV response to tension remains relevant^66,67^. The relationship between tension and squared SWV is displayed in Fig. 3f.

### Neurosurgical case series

#### Participants and surgical procedure

From January to December 2022, six consecutive patients were prospectively enrolled in the observational study. Inclusion criteria consisted of patients presenting with neurological symptoms and deficits despite one or more conventional detethering operations, diagnosed with recurrent TCS, and scheduled to undergo a PVCSO intervention. All patients presented with lower extremity weakness and decreased sensation, as well as bladder deficits. PVCSO is a treatment option in cases of failed detethering and involves the removal of a section of bone (T12 in this series) to reduce tension across the cord^53^. Informed written consent was obtained for the surgical procedure and intraoperative ultrasound, adherent to the approved John Hopkins University Institutional Review Board protocol (#00273900). Our work aligned with the STrengthening the Reporting of Observational Studies in Epidemiology (STROBE) reporting guidelines and best practices^68^. All patients underwent successful and standard PVCSO procedures with observational ultrasound acquisitions. The mean height reduction to the spinal column in previous cases published by our institution was 23.4 ± 2.7 mm, shown to reduce spinal cord tension in cadavers^53,69^.

#### Intraoperative ultrasound collection and processing

The surgical site was irrigated with saline solution during each of the three ultrasound imaging checkpoints: before shortening (PRE), at the midpoint during shortening (MID), and after completion of the full shortening (FULL). A Canon i18LX5 ultrasound transducer was manually held in the sagittal plane over the spinal cord. The probe was adjusted by the neurosurgeons to optimize the focus, positioning the central canal of the cord within the field of view. Three to six ultrasound acquisitions were collected at each checkpoint. A sample propagation map and shear wave heatmap from each checkpoint of each case are included in Supplementary Fig. 1.

Data were reviewed by radiologists and the chief ultrasound technologist at our hospital (*N* = 3). Only given the B-mode images and blinded to the shear wave data, the team tagged sections of each image as in-plane or out-of-plane based on whether the central canal was visible. If the canal was not detectable in a section of the spinal cord, that region was discarded from further analysis. If the canal was entirely undetectable in an image, that acquisition was completely rejected. The bulk of the data was deemed acceptable for systematic processing on the Canon system. Sub-samples were captured across the spinal cord. The smallest circular sub-region of interest tool on the Canon system (size 1) was used to methodically collect columns of datapoints along the cord for each image (which amounted to 7000+ datapoints across the 6 cases). A schematic of this data extraction is illustrated in Supplementary Fig. 2a, b. All ultrasound images with fewer than 30 datapoints were discarded due to poor acquisition quality.

The second stage of processing (beyond the Canon system) measured the curvature of the spinal cord. Shear waves are tracked linearly, and their propagation can be disrupted by tissue boundaries and fluids (e.g., saline and cerebrospinal fluid). Accordingly, anatomical positioning can disrupt acquisition quality when the Euclidean path from end-to-end of the imaged spinal cord traverses the confines of the cord (Supplementary Fig. 2c). If the final imaging checkpoint (FULL) met these exclusion criteria, the imaging checkpoint before it (MID) was utilized as the POST intervention data. To make this determination, the shear wave propagation map of each acquisition was first exported as a Digital Imaging and Communication in Medicine (DICOM) file and analyzed in MicroDicom (version 3.9.5, Sofia, Bulgaria). The relative curvature was also measured (Supplementary Fig. 2d) and shown (Supplementary Fig. 3a).

We define relative curvature as the amount of excess (indirect) length that the spinal cord travels from point A to point B as a percentage of that direct A to B distance. Relative curvature is used to quantify the anatomical geometry of the spinal cord during and after shortening, in which slack arises when tension is released. For PVCSO procedures, one of the major decisions that clinicians must make is how much they will shorten the patient’s spinal column. If the column is not shortened enough, the tension (and thus symptoms) could remain. Conversely, if shortened too much, additional complications might arise. In conjunction with SWV, relative curvature values should be analyzed in future work as potential predictors of long-term patient outcomes and for the utility of TAUT for intraoperative feedback.

### Statistics and reproducibility

**Fig. 4:**
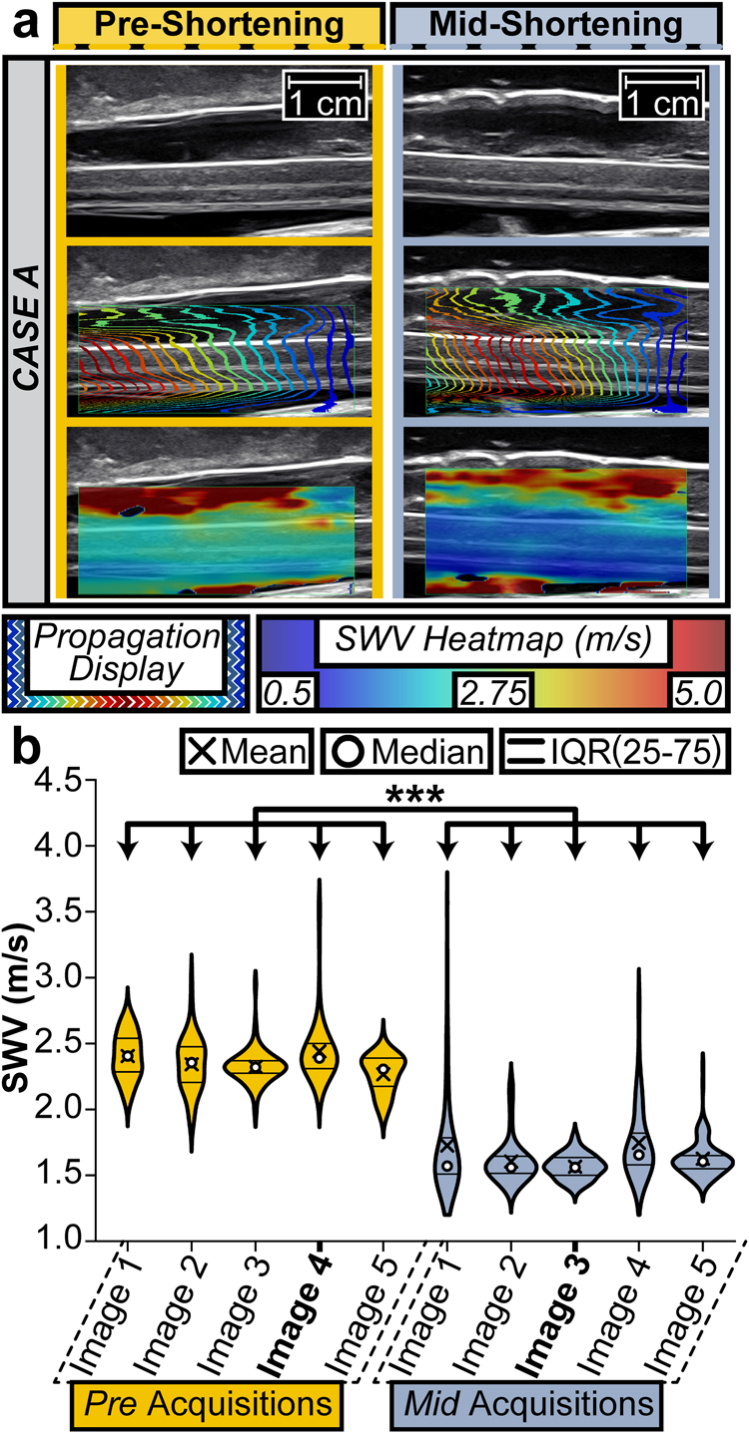
Intraoperative use of TAUT for spinal cord stretch differentiation. **a** A posterior vertebral column subtraction osteotomy (PVCSO) surgery was conducted to alleviate tension across the spinal cord of a patient diagnosed with recurrent TCS. By decreasing the height of the patient via bone removal, the distance that the cord stretches should be reduced. To intraoperatively evaluate the presence of (and changes in) tension, ultrasound was utilized to induce and record shear waves. Images from before (the fourth pre-acquisition) and midway through (the third mid-acquisition) the procedure are shown for Case A. Both columns include a single B-mode anatomical capture, shear wave propagation display, and the corresponding SWV heatmap. **b** SWV datapoints from all ultrasound image acquisitions pre- and mid-shortening are plotted from Case A. The violin plots and the corresponding interquartile ranges (IQR) are displayed to help visualize the distribution of SWV sub-sampling which compose our dataset. The following number of sub-samples are present from pre-image one to mid-image five: 76, 60, 72, 84, 76, 116, 72, 64, 96, and 112. The decrease in spinal cord tension between surgical timepoints was captured by TAUT (***p < 0.001). Additional information regarding statistics including p-values can be found in Supplementary Data 1.

The intraoperative data was sub-divided with respect to 3 attributes: patient case (A through F), stage of operation (PRE, MID, or FULL), and each image acquisition number in the sub-session (1−6). We compared the measurements of the cord under tension (PRE for all cases) to the relaxed cord (POST) after shortening, either MID or FULL based on the curvature exclusion criteria. The change in SWV between PRE and POST for each case was analyzed using the Student’s t-test of unequal sample sizes and unequal variances with *α* = 0.001. To demonstrate the utility of TAUT, a single-factor analysis of variance (ANOVA) with *α* = 0.05 was used in Fig. 4b. With a significant difference detected, we applied a Tukey-Kramer post hoc analysis for unequal sample sizes with a critical value *α* = 0.001 for the image comparisons.

SWV was analyzed as a binary diagnostic tool or differentiator between stretched and healthy spinal cords. A receiver operating characteristic (ROC) curve was created and analyzed in Origin (version 2022b). The area under the curve (AUC) (i.e., *c*-statistic) and the corresponding 95% confidence interval are included. The SWV value that maximized Youden’s index was analyzed in parallel with the simulation-derived SWV value. The sensitivity and specificity of both SWV thresholds were calculated.

### Reporting summary

Further information on research design is available in the Nature Portfolio Reporting Summary linked to this article.

## Results

The data supported the hypothesis in all three sub-studies: TAUT differentiated stretched from healthy spinal cords, aiding in direct TCS evaluations. Ultrasound-induced shear waves allowed for a successful quantification of stretch.

### Finite element simulation

In the simulated spinal cord model, shear waves propagated faster when the cord was under tension (Fig. 2). The squared SWV from each loading condition is shown in Fig. 2c. The SWV was 4.16 m/s at a maximum tension of 256 g, while only 1.39 m/s when fully relaxed. This 67% decrease in SWV demonstrated the direct influence of and sensitivity to tension. The simulated shear wave propagation through these tethered and relaxed cords can be seen in Supplementary Videos 1 and 2, respectively. Utilizing the entire finite element dataset, tension and squared SWV exhibited a linear relationship as confirmed by linear regression (*R*^2^ = 0.984). The best-fit line for this relationship had a slope of 0.058 m^2^ s^−2^ g^−1^ which intersected the zero-force, “simulation baseline” state at 1.76 m/s. This baseline is the expected threshold between healthy and stretched spinal cords.

### Cadaveric Benchtop Study

The elongation of the spinal cord was visualized via ultrasound imaging as depicted in Supplementary Video 3 while the conus medullaris was pulled caudally. The linear relationship between tension and squared SWV was confirmed via a linear regression (*R*^2^ = 0.951; Fig. 3). As expected, the tissue properties of a cadaver elevated all SWV values^66,70^. The maximum load average SWV was 5.09 m/s. Despite the upward shift in the squared SWV-tension best-fit line, the slope was 0.053 m^2^ s^−2^ g^−1^. Additionally, the anterior half of the spinal cord was observed to have a faster SWV regardless of the tensile force applied.

### Neurosurgical case series

**Fig. 5:**
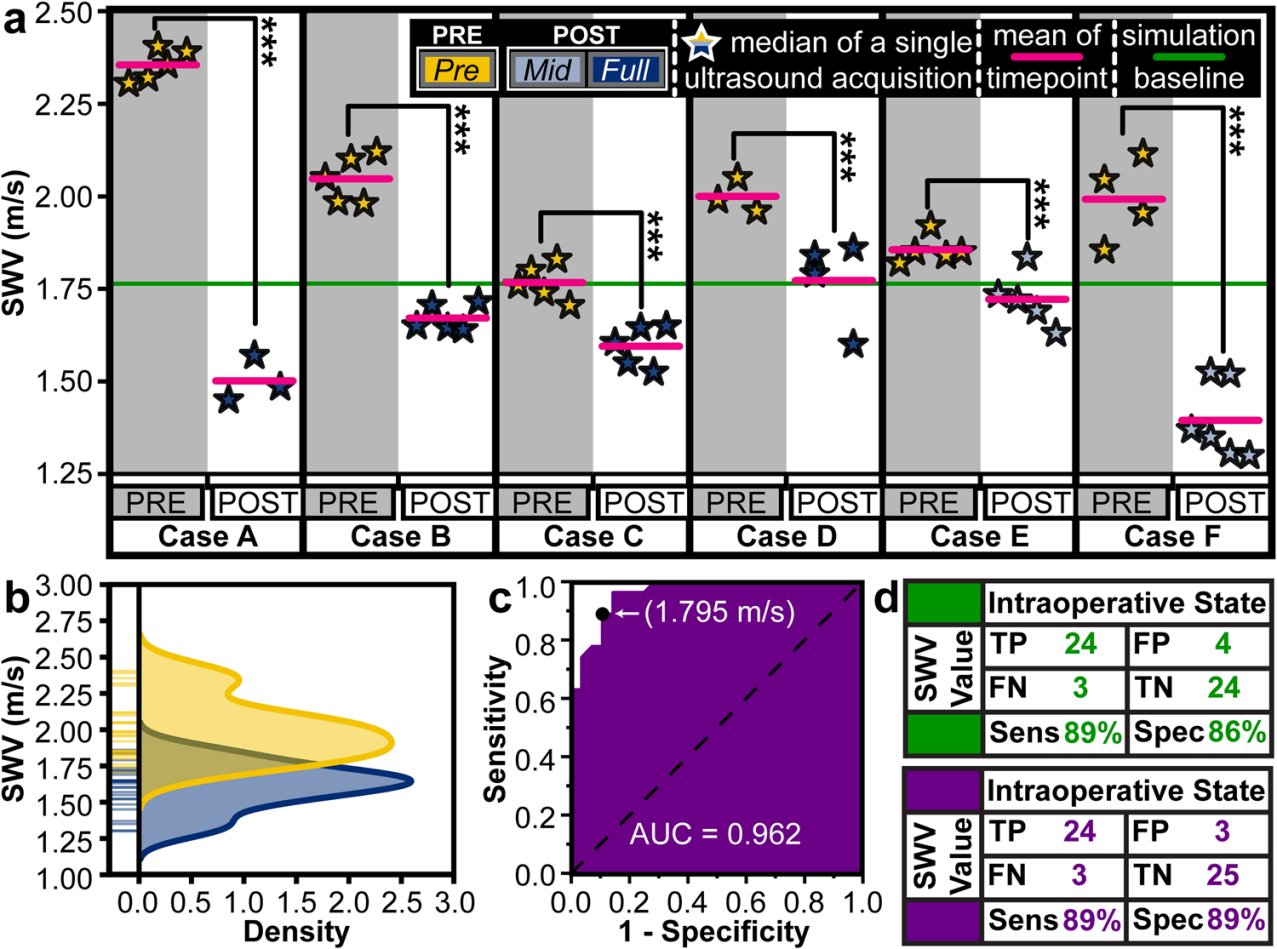
Intraoperative case series successfully demonstrated the utility of TAUT to detect tensile differences across spinal cords. **a** Six patients with recurrent TCS underwent a PVCSO procedure aimed at reducing spinal cord stretch. Ultrasound-induced shear waves were captured during the procedure. MID-shortening was used as the POST intervention when FULL-shortening did not meet the inclusion criteria, as shown in Supplementary Fig. 2c. The green line serves as a reference for the expected threshold of a healthy spinal cord as modeled by the finite element simulation. The median SWV of each image acquisition was plotted as a star. The magenta line marks the mean value of the image medians. A statistically significant difference in SWV was observed during each case (PRE-POST) and when pooled across the entire neurosurgical series (***p < 0.001). Statistical calculations including p-values can be found in Supplementary Data 1. **b** The capability of a single ultrasound image SWV to detect the presence of tension is depicted and analyzed. The yellow (PRE) and blue (POST) ticks are the combined median image acquisition values from all neurosurgical cases. The SWV distributions of both intraoperative states are plotted. **c** From this data, the receiver operator characteristic (ROC) and corresponding area under the curve (AUC) are plotted for the SWV threshold which maximizes Youden’s index. **d** The purple chart quantifies the sensitivity and specificity of the TAUT diagnostic capabilities with the ROC-derived SWV threshold. Similarly, the green chart quantifies the utility of TAUT with the simulation-derived SWV value. TP true positive, FP false positive, FN false negative, TN true negative.

The FULL acquisitions in cases A, B, C, and D qualified as POST data. Due to curvature exclusion criteria, MID acquisitions from cases E and F were classified as POST data. Shear wave heatmap color changes indicative of tension reduction were often visually observed intraoperatively (Fig. 4a). The distribution of sub-samples and statistical significance of data across one of these imaging checkpoints is shown in Fig. 4b. Notably, a decrease in SWV (PRE to POST) was detected during all PVCSO procedures as shown in Fig. 5a (*p* < 0.001).

To evaluate the preliminary utility of TAUT as a diagnostic tool, the median SWV of each acquisition was compared to the simulation-derived threshold for the presence of tension (1.76 m/s). A true positive tension diagnosis was defined as a SWV above the threshold before shortening. The PRE mean SWV values before each case exceeded the simulated threshold (100% tension detection). Similarly, successful diagnosis of a treated (relaxed) spinal cord was defined by a SWV below 1.76 m/s. Of the 55 images taken across the PRE and POST images, 48 of the acquisitions aligned with the simulated threshold expectations (PRE above the threshold and POST below the threshold). As a single-image diagnostic tool, the accuracy was 87.3%. When ultrasound images in the same imaging checkpoint were averaged, the diagnosis aligned with the expectation all but once (91.7%, 11 out of 12). With the simulation-derived threshold, the single-image sensitivity and specificity were 89% and 86%, respectively (Fig. 5d).

Without insight into the simulation data, the diagnostic utility of TAUT was analyzed. The ROC AUC was 96.2% (95% Confidence Interval: 92%–100%). The SWV value that maximized Youden’s index was 1.795 m/s (Fig. 5c). This threshold had a single-image sensitivity and a single-image specificity of 89% (Fig. 5d).

## Discussion

TCS produces substantial neurological deficits and is often refractory to conservative treatment and surgical detethering. Unfortunately, no modalities are currently employed to quantify the amount of tension across the cord, which could aid in the preoperative diagnosis, intraoperative workflow, and postoperative monitoring for recurrence.

We report an ultrasound-based approach to quantify spinal cord tension. A finite element simulation, a human cadaveric study, and 6 human intraoperative cases confirmed the ability of TAUT to detect differences between a stretched and a relaxed spinal cord. This ultrasound metric confirmed that a successful PVCSO procedure modulates the underlying pathophysiology (tension) without requiring surgical removal of the tissue causing tethering.

The finite element simulation was purposefully simplistic to isolate the SWV-tension relationship. Although the computationally measured tension threshold of 1.76 m/s aligned well with the intraoperative data, we do not suggest the widespread adoption of this value in the clinic until further studies are performed. To increase reliability and accuracy, we recommend using patient-specific information and revisiting compositional assumptions in future simulations. For example, additional simulations could account for the complexities and variation of patient anatomy (e.g., the geometry of the spinal cord, reflective artifacts from bone, and shear wave boundary artifacts at the fluid-tissue interface). Our simulation was built to mimic the commercial system which was utilized in the cadaveric and intraoperative sub-studies. Consequently, there were linear elastic assumptions and several advanced shear wave phenomena such as boundary condition disruptions (e.g., Scholte waves^71^) were ignored. The Canon ultrasound transducer has proprietary components, and therefore, extrapolations were made from open-source commercial probes to mimic the clinical setup (Supplementary Table 1). This approach enabled us to study the SWV response as a function of tension in the spinal cord (Supplementary Videos 1 and 2) in a controlled and isolated manner.

The cadaveric benchtop model bridged the gap between the simulation and intraoperative case series. This sub-study validated the linear relationship between tension and squared SWV in a human spinal cord. A live human spinal cord has drastically different acoustic and tissue properties than a fresh-cooled cadaver^70,72^. Due to this, the absolute SWV in the baseline (non-stretched) cadaveric spinal cord was almost twice that of the non-stretched simulation and unstretched intraoperative cases. Despite this, the slope of the cadaveric squared SWV-tension best-fit line (0.053 m^2^ s^−2^ g^−1^) was similar to the finite element simulation fit (0.058 m^2^ s^−2^ g^−1^). Notably, a faster SWV was observed in the anterior region of the cadaveric cord, distal to the transducer (Supplementary Video 3). This non-uniformity may be attributed to boundary conditions, Scholte waves, or tissue anisotropy^71^. While anatomical section-by-section SWV analysis could provide insights, our approach successfully represented the applied tension. Future work could couple TAUT with rigorous ex vivo tensile testing to best understand and validate the spinal cord force dynamics^73^.

Our human case series was, to the best of our knowledge, the first known intraoperative assessment of spinal cord tension with shear wave elastography or Tensography (i.e., TAUT). Other ultrasound modalities have previously been utilized in PVCSO but not to quantify tension^74^. While tracking changes in pulsatility, blood flow, and electrophysiological recordings might provide insights, they are secondary indicators to tension, the cause of TCS. In our study (cases A and B), relaxation of the cord after shortening was not visually detectable from B-mode images, further demonstrating the need for TAUT (Fig. 4a).

Throughout our studies and for future investigations, it is important to be mindful of SWV fluctuations from factors including the machine utilized and depth of imaging^75,76^. All sub-samples were pooled to create a single ultrasound acquisition SWV value. The use of small circular sub-regions of interest to sample the entire field was inefficient, non-modular, and could be prone to operator error. Future investigations may analyze the entirety of the acquisition by assessing the SWV as a function of anatomical position. Furthermore, these regions of notably high curvature were observed to have an increased SWV (Supplementary Fig. 3a). A more in-depth spatial analysis may reveal the physiological impact of locally elevated SWV.

All patients improved functionally after their shortening procedure, including patient E with an increased SWV in the FULL checkpoint. An ideal PVCSO should remove the underlying tension while minimizing any excess removal of bone^65,77^. TAUT may be capable of providing meaningful intraoperative feedback to optimize the amount of shortening that should be performed. Future work may analyze long-term patient outcomes with the intraoperatively measured SWV decreases to enable clinically relevant insights to be drawn from TAUT.

Noninvasive shear wave imaging after PVCSO intervention is an uninvestigated and potentially helpful tool. A single imaging plane can capture TAUT measurements even if obstructions from hardware or ossification are present. If these acoustic windows are suitable for noninvasive TAUT imaging, critical information regarding the state of tension over time might be obtained. Expansion of this technology—especially in the intraoperative setting—into larger cohorts is warranted, as the benefits of TAUT were demonstrated across all 6 cases.

In addition to allowing for greater PVCSO data collection, SWV acquisitions in other spine cases may provide a broader context for TAUT. To further explore healthy SWV ranges, future studies can acquire data in non-TCS neurosurgical interventions. For example, although seemingly healthy adult patients are unlikely to undergo surgery, relevant cases for non-stretched TAUT acquisitions may include select extradural tumor excision or dorsal root entry zone lesioning procedures.

The ability to gauge forces across tissues or organs beyond the spinal cord is a growing frontier of medicine. For example, assessing the loading of musculoskeletal system tissues with shear waves is an emerging field, as the ability of the tissue to withstand and apply forces is a primary function. Musculoskeletal tissues including tendons undergo dynamic tensile changes from a stretched to a relatively relaxed state; the change in shear wave measurements can be captured in both states to aid in performance evaluations. Similarly, our TAUT case series captured changes in SWV from a stretched to a relaxed state. As the deployment and uses of TAUT expand, incorporating parallel advances in the field beyond the central nervous system will further help refine the utility of SWV for spinal cord applications.

In summary, TAUT provides clinicians with readily available, quantitative insights regarding the tensile loading across a patient’s spinal cord. We illustrate that TAUT can help evaluate TCS and confirm a reduction of tension intraoperatively. In addition to tracking these immediate changes in tension with TAUT, the approach could be utilized to compliment studies on the developing spinal cord^78^. Our findings bring promise to the idea of noninvasively diagnosing patients with an existing acoustic window. Eligible patients may include the pediatric population (without a fully ossified posterior spine) and individuals with a previous laminectomy. Additional refinements to establish absolute and relative SWV thresholds of tension will likely help improve outcomes and quality of life in patients with TCS.

### Supplementary information

Supplementary Data 1

Supplementary Information

Supplementary Video 1

Supplementary Video 2

Supplementary Video 3

Reporting Summary

Description of Additional Supp Files

## Data Availability

The numerical data obtained during the three sub-studies and displayed in Figs. 2–5 are included in Supplementary Data 1. Any additional information about the study can be obtained from the corresponding author upon reasonable request.
