# Supplementary material for: Tethered spinal cord tension assessed via ultrasound elastography in computational and intraoperative human studies
Source: Commun Med (Lond). 2024 Jan 5;4:4. doi: 10.1038/s43856-023-00430-6 (PMC10770351; doi:10.1038/s43856-023-00430-6)
Supplement: Supplementary file 2 — Supplementary Information [file 43856_2023_430_MOESM2_ESM.pdf]

Supplementary Information

Tethered spinal cord tension assessed via ultrasound elastography in computational and intraoperative human studies

Max J. Kerensky[1,2], Abhijit Paul[3], Denis Routkevitch[1,2,4], Andrew M. Hersh[2,4], Kelley M. Kempski-Leadingham[2,4], A. Daniel Davidar[2,4], Brendan F. Judy[4], Joshua Punnoose[2,4], Autumn Williams[1], Avisha Kumar[2,5], Kurt Lehner[4], Beth Smith[2,6], Jennifer K. Son[2,6], Javad R. Azadi[2,6], Himanshu Shekhar[7], Karla P. Mercado-Shekhar[3], Nitish V. Thakor[1,2,5,8], Nicholas Theodore[1,2,4,9,10], Amir Manbachi[1,2,4,5,11,12]*

[1]Department of Biomedical Engineering, Johns Hopkins University School of Medicine, Baltimore, MD, USA

[2]HEPIUS Innovation Laboratory, Johns Hopkins University School of Medicine, Baltimore, MD, USA

[3]Discipline of Biological Engineering, Indian Institute of Technology Gandhinagar, Gujarat, India

[4]Department of Neurosurgery, Johns Hopkins University School of Medicine, Baltimore, MD, USA

[5]Department of Electrical and Computer Engineering, Johns Hopkins University, Baltimore, MD, USA

[6]Department of Radiology and Radiological Science, Johns Hopkins University School of Medicine, Baltimore, MD, USA

[7]Discipline of Electrical Engineering, Indian Institute of Technology Gandhinagar, Gujarat, India

[8]Department of Neurology, Johns Hopkins University School of Medicine, Baltimore, MD, USA

[9]Department of Orthopaedic Surgery, Johns Hopkins University School of Medicine, Baltimore, MD, USA

[10]Department of Pediatrics, Johns Hopkins University School of Medicine, Baltimore, MD, USA

[11]Department of Anesthesiology and Critical Care Medicine, Johns Hopkins University School of Medicine, Baltimore, MD, USA

[12]Department of Mechanical Engineering, Johns Hopkins University, Baltimore, MD, USA

***Correspondence:** Amir Manbachi, PhD (Amir.Manbachi@jhu.edu)
Johns Hopkins Bayview Medical Center, Alpha Center 116, 5210 Eastern Ave, Baltimore, MD 21224

Supplementary Table 1. Finite element simulation parameters of the medium and linear array transducer.

**Medium parameters**			
	**Spinal cord model**		**Aqueous Solution**
Properties	Lateral direction	Axial & elevation direction	
Young's modulus (Pa)	5996 [1–3]	2998 [2,4]	2.998×10^{-6}
Poisson's ratio	0.499 [5–7]	0.499 [5–7]	0.499
Shear modulus (Pa)	2000	1000	10^{-6} [8]
Density (kg/m^3)	1075 [9,10]	1075 [9,10]	1000
Attenuation coefficient (dB/cm/MHz)	1 [11]	1 [11]	0.002 [12]
Sound speed (m/s)	1542 [10]	1542 [10]	1500 [13]
**Linear array transducer parameters**			
Center frequency (MHz)	11.5	Number of elements	256
Sampling frequency (MHz)	100	F number	1.04
Width (mm)	0.053	Bandwidth ratio	0.73
Kerf (mm)	0.007	Acoustic impulse push duration (µs)	200
Focus (mm)	15	Peak intensity at the focus (W/cm^2)	1000 [14–16]
Height (mm)	7		

Supplementary Methods Additional information regarding the finite element simulation.

The acoustic impulse is the acoustic radiation force that was computed by the following formula:

$$\vec{F} = \frac{2 * \alpha * \vec{I}}{c}$$

where α denotes the acoustic attenuation coefficient of the simulated tissue medium, $\vec{I}$ denotes the simulated acoustic intensity, and *c* denotes the longitudinal sound speed of the simulated tissue medium[14]. This acoustic radiation force impulse was applied onto the medium as a body force/load for 0.08 ms. The intensity *I* is the spatial peak temporal average intensity, which was computed using the following equation:

$$I = \frac{P^2}{2 * Z * T}$$

where *P* denotes the simulated acoustic pressure field (obtained using Field II)[17,18], *Z* denotes acoustic impedance of the simulated tissue medium, and *T* denotes the total time of excitation pulse or push beam. In the present study, we considered a single push beam; therefore, the spatial peak temporal average and spatial peak pulse average will be identical.

The acoustic radiation force was then converted into an impulsive body force by multiplying with an impulsive time function in COMSOL. The impulsive body force remains 'on' for 0.08 ms, and then it remains 'off' for the rest of the shear wave propagation period of 2.3 ms in the simulation model.

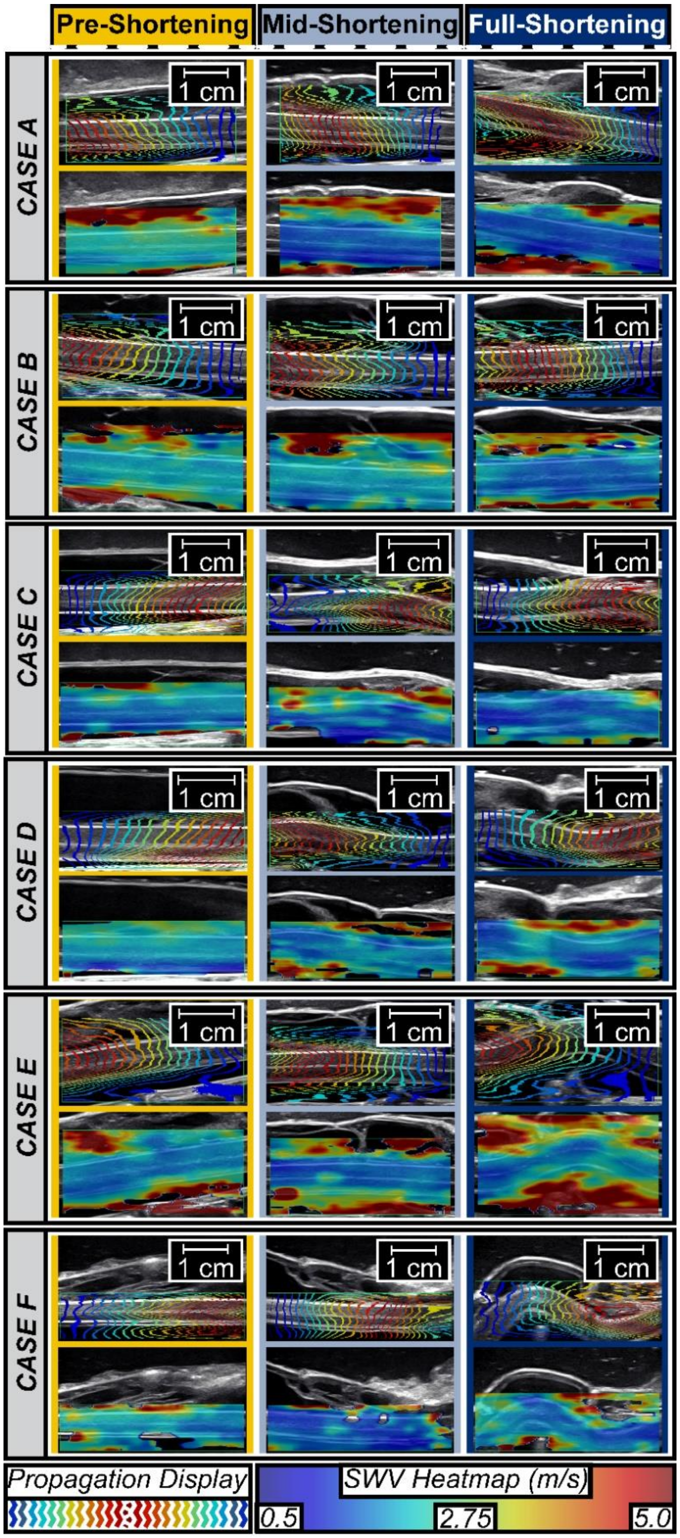

Supplementary Figure 1. Intraoperative progression of "Tethered cord Assessment with Ultrasound Tensography" (TAUT) in the posterior vertebral column subtraction osteotomy (PVCSO) case series. A single image acquisition from each timepoint is displayed for each of the 6 cases. The propagation displays and shear wave velocity (SWV) heatmaps are captured and visible during the surgical procedures.

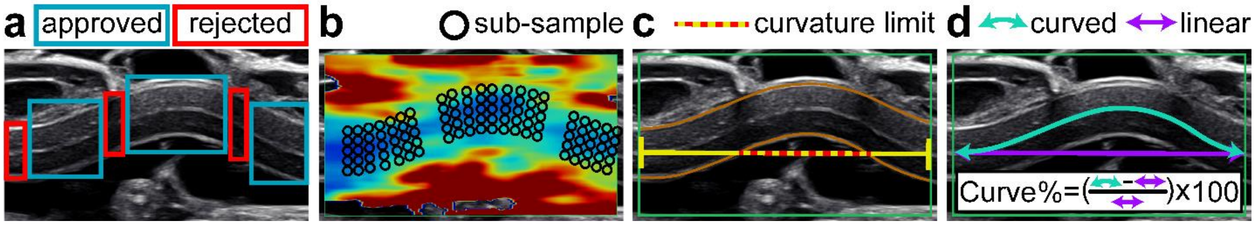

Supplementary Figure 2. Schematic of tagging, sampling, and measuring ultrasound data from the intraoperative case series. a Radiologists and sonographers were blinded to shear wave data and tagged B-mode images based on the visibility of the central canal to ensure all measurements were reliably in-plane. **b** On the commercial ultrasound system, sub-samples were methodically extracted from approved regions. A schematic displays these virtual sub-samples on a heatmap. The actual extractions occurred on the propagation display without SWV values accessible. **c** Surgical timepoints exceeding the curvature limit (red dotted line) were excluded as the fluid-tissue interface becomes a factor. In cases with this confounding variable, the MID-shortening timepoint was utilized as the POST-intervention (as seen in Fig. 5 and associated analyses). **d** The relative curve percentage as displayed in Supplementary Figure 3 was measured by subtracting the end-to-end linear length (purple) from the curved length of the central canal (cyan), divided by the linear length, and then multiplied by 100.

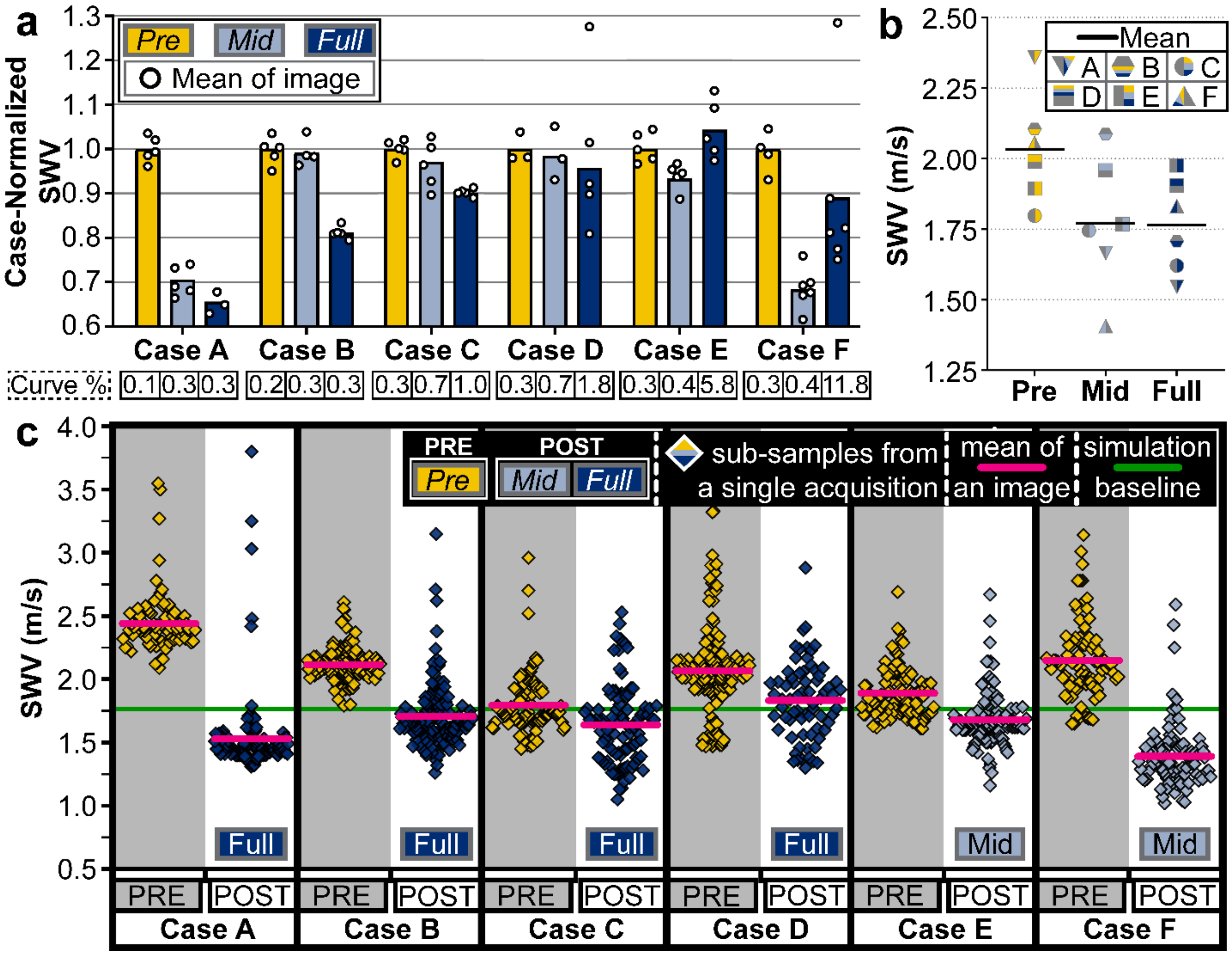

Supplementary Figure 3. Ultrasound-induced shear wave data visualizations from the PVCSO case series. a Case-normalized box plots depict the relative SWV changes across each procedure. Below each plot is the average relative curve percentage (Supplementary Figure 2d) at each surgical timepoint. The rebound of SWV closely aligned with an increased curve percentage. **b** The average SWV at each surgical timepoint across the case series were pooled and plotted. **c** The sub-samples of a single image acquisition are shown (for visualization purposes) for each of the cases pre- and post-shortening (as defined by Supplementary Figure 2c). The green line serves as a reference for the expected threshold of a healthy spinal cord as modeled in the finite element simulation.

Supplementary References

1. Bartlett, R. D., Choi, D. & Phillips, J. B. Biomechanical properties of the spinal cord: implications for tissue engineering and clinical translation. *Regenerative Med* **11**, 659–673 (2016).
2. Nishida, N. *et al.* Tensile mechanical analysis of anisotropy and velocity dependence of the spinal cord white matter: a biomechanical study. *Neural Regen Res* **16**, 2557–2562 (2021).
3. Jin, C. *et al.* Dynamic changes in mechanical properties of the adult rat spinal cord after injury. *Acta Biomater* **155**, 436–448 (2023).
4. Ozawa, H., Matsumoto, T., Ohashi, T., Sato, M. & Kokubun, S. Mechanical properties and function of the spinal pia mater. *J Neurosurg Spine* **1**, 122–127 (2004).
5. Hosey, R. R. A homeomorphic finite-element model of impact head injury. (Tulane University, 1981).
6. Eskandari, F., Rahmani, Z. & Shafieian, M. The effect of large deformation on Poisson's ratio of brain white matter: An experimental study. *Proc Inst Mech Eng H* **235**, 401–407 (2021).
7. Ting, T. C. T. & Chen, T. Poisson's ratio for anisotropic elastic materials can have no bounds. *Q J Mech Appl Math* **58**, 73–82 (2005).
8. Apakashev, R. A. & Pavlov, V. V. Determination of the shear strength and modulus of water at low flow velocities. *Fluid Dynamics* **32**, 1–4 (1997).
9. Masuda, S., Mori, A., Mizonishi, S. & Tashiro, R. Creation of an Analytical Model of Spinal Cord Cooling by Epidural Catheter for Preventing Paraplegia. *Cureus* (2021) doi:10.7759/cureus.20430.
10. Hasgall, P. *et al.* Tissue Properties Database V4.1. *IT'IS Foundation* 10.13099/VIP21000-04-1 (2022) doi:10.13099/VIP21000-04-1.
11. Kremkau, F. W., Barnes, R. W. & McGraw, C. P. Ultrasonic attenuation and propagation speed in normal human brain. *J Acoust Soc Am* **70**, 29–38 (1981).
12. Shankar, H. & Pagel, P. S. Potential adverse ultrasound-related biological effects: A critical review. *Anesthesiology* **115**, 1109–1124 (2011).
13. Afaneh, A., Alzebda, S., Ivchenko, V. & Kalashnikov, A. N. Ultrasonic Measurements of Temperature in Aqueous Solutions: Why and How. *Physics Research International* **2011**, 1–10 (2011).
14. Palmeri, M. L., Qiang, B., Chen, S. & Urban, M. W. Guidelines for Finite-Element Modeling of Acoustic Radiation Force-Induced Shear Wave Propagation in Tissue-Mimicking Media. *IEEE Trans Ultrason Ferroelectr Freq Control* **64**, 78–92 (2017).
15. Nightingale, K. Acoustic Radiation Force Impulse (ARFI) Imaging: a Review. *Curr Med Imaging Rev* **7**, 328–339 (2011).
16. Davies, G. & Koenen, M. Acoustic radiation force impulse elastography in distinguishing hepatic haemangiomata from metastases: preliminary observations. *Br J Radiol* **84**, 939–943 (2011).
17. Jensen, J. A. & Svendsen, N. B. Calculation of pressure fields from arbitrarily shaped, apodized, and excited ultrasound transducers. *IEEE Trans Ultrason Ferroelectr Freq Control* **39**, 262–267 (1992).
18. Jensen, J. A. Field: A program for simulating ultrasound systems. *Med Biol Eng Comput* **34**, 351–353 (1997).
