# Supplementary material for: Tethered spinal cord tension assessed via ultrasound elastography in computational and intraoperative human studies
Source: Commun Med (Lond). 2024 Jan 5;4:4. doi: 10.1038/s43856-023-00430-6 (PMC10770351; doi:10.1038/s43856-023-00430-6)
Supplement: Supplementary file 6 — Reporting Summary [file 43856_2023_430_MOESM6_ESM.pdf]

Corresponding author(s): Amir Manbachi, PhD

Last updated by author(s): Dec 4, 2023

Reporting Summary

Nature Portfolio wishes to improve the reproducibility of the work that we publish. This form provides structure for consistency and transparency in reporting. For further information on Nature Portfolio policies, see our Editorial Policies and the Editorial Policy Checklist.

Statistics

For all statistical analyses, confirm that the following items are present in the figure legend, table legend, main text, or Methods section.

n/a	Confirmed	
☐	☒	The exact sample size (n) for each experimental group/condition, given as a discrete number and unit of measurement
☐	☒	A statement on whether measurements were taken from distinct samples or whether the same sample was measured repeatedly
☐	☒	The statistical test(s) used AND whether they are one- or two-sided<br>*Only common tests should be described solely by name; describe more complex techniques in the Methods section.*
☒	☐	A description of all covariates tested
☒	☐	A description of any assumptions or corrections, such as tests of normality and adjustment for multiple comparisons
☐	☒	A full description of the statistical parameters including central tendency (e.g. means) or other basic estimates (e.g. regression coefficient) AND variation (e.g. standard deviation) or associated estimates of uncertainty (e.g. confidence intervals)
☐	☒	For null hypothesis testing, the test statistic (e.g. F, t, r) with confidence intervals, effect sizes, degrees of freedom and P value noted<br>*Give P values as exact values whenever suitable.*
☒	☐	For Bayesian analysis, information on the choice of priors and Markov chain Monte Carlo settings
☒	☐	For hierarchical and complex designs, identification of the appropriate level for tests and full reporting of outcomes
☒	☐	Estimates of effect sizes (e.g. Cohen's d, Pearson's r), indicating how they were calculated

Our web collection on statistics for biologists contains articles on many of the points above.

Software and code

Policy information about availability of computer code

Data collection	Aplio i800 in 2D-SWE mode with an i18LX5 transducer (Canon Medical Systems, Otawara, Japan), COMSOL Multiphysics
Data analysis	Origin (version 2022b), MATLAB (version R2021a), Excel (Microsoft; USA)

For manuscripts utilizing custom algorithms or software that are central to the research but not yet described in published literature, software must be made available to editors and reviewers. We strongly encourage code deposition in a community repository (e.g. GitHub). See the Nature Portfolio guidelines for submitting code & software for further information.

Data

Policy information about availability of data

All manuscripts must include a data availability statement. This statement should provide the following information, where applicable:

- Accession codes, unique identifiers, or web links for publicly available datasets
- A description of any restrictions on data availability
- For clinical datasets or third party data, please ensure that the statement adheres to our policy

The data and supporting findings from this work are in the main text and Supplementary Information, with underlying data utilized from the three sub-studies in Supplementary Data 1. Any additional information is available from the corresponding author upon reasonable request.

Research involving human participants, their data, or biological material

Policy information about studies with human participants or human data. See also policy information about sex, gender (identity/presentation), and sexual orientation and race, ethnicity and racism.

Reporting on sex and gender	This was a small case series (3 male / 3 female) and was analyzed across the full group of six patients.
Reporting on race, ethnicity, or other socially relevant groupings	All patients had recurrent tethered cord. A small population with a rare neurological condition that was not adequately addressed through one or more detethering procedure.
Population characteristics	Patient ages ranged from 34-51 years. Patient weights ranged from 54.6-104.3 kg.
Recruitment	From January to December 2022, six consecutive patients were prospectively enrolled in the observational study. Inclusion criteria consisted of patients presenting with neurological symptoms and deficits despite one or more conventional detethering operations, diagnosed with recurrent TCS and scheduled to undergo a PVCSO intervention. All patients presented with lower extremity weakness and decreased sensation, as well as bladder deficits.
Ethics oversight	Informed consent was obtained for the surgical procedure and for intraoperative ultrasound, adherent to the approved John Hopkins University. Institutional Review Board protocol (#00273900). We complied with and were mindful of all guidelines, ethical standards, and policies.

Note that full information on the approval of the study protocol must also be provided in the manuscript.

Field-specific reporting

Please select the one below that is the best fit for your research. If you are not sure, read the appropriate sections before making your selection.

☒ Life sciences ☐ Behavioural & social sciences ☐ Ecological, evolutionary & environmental sciences

For a reference copy of the document with all sections, see nature.com/documents/nr-reporting-summary-flat.pdf

Life sciences study design

All studies must disclose on these points even when the disclosure is negative.

Sample size	A single TCS model was created in an adult human cadaver with no known history of prior surgeries and devoid of confounding spine pathology (State Anatomy Board of Maryland, pre-registered donation with informed consent for medical research).
Data exclusions	No data was excluded.
Replication	Three measurements were taken at each condition. The steps to run this study are in the manuscript.
Randomization	Not relevant. Tension must be induced incrementally.
Blinding	Not relevant as quantification is based on anatomical boundaries.

Reporting for specific materials, systems and methods

We require information from authors about some types of materials, experimental systems and methods used in many studies. Here, indicate whether each material, system or method listed is relevant to your study. If you are not sure if a list item applies to your research, read the appropriate section before selecting a response.

Materials & experimental systems

n/a	Involved in the study	
☒	☐	Antibodies
☒	☐	Eukaryotic cell lines
☒	☐	Palaeontology and archaeology
☒	☐	Animals and other organisms
☒	☐	Clinical data
☒	☐	Dual use research of concern
☒	☐	Plants

Methods

n/a	Involved in the study	
☒	☐	ChIP-seq
☒	☐	Flow cytometry
☒	☐	MRI-based neuroimaging
