# Supplementary material for: Tethered spinal cord tension assessed via ultrasound elastography in computational and intraoperative human studies
Source: Commun Med (Lond). 2024 Jan 5;4:4. doi: 10.1038/s43856-023-00430-6 (PMC10770351; doi:10.1038/s43856-023-00430-6)
Supplement: Supplementary file 7 — Description of Additional Supp Files [file 43856_2023_430_MOESM7_ESM.docx]

Description of Additional Supplementary Files

**File name:** Supplementary Video 1

**Description:**Shear wave propagation simulation with maximum tension

**File name:** Supplementary Video 2

**Description:**Shear wave propagation simulation without tension

**File name:** Supplementary Video 3

**Description:**Cadaveric progression of TAUT

**File name:** Supplementary Data 1

**Description:** Data from the three sub-studies
